# Survival in patients with non-metastatic breast cancer treated with adjuvant trastuzumab in clinical practice

**DOI:** 10.1186/s40064-016-2008-9

**Published:** 2016-03-31

**Authors:** Christopher M. Gallagher, Kenneth More, Anthony Masaquel, Tripthi Kamath, Annie Guerin, Raluca Ionescu-Ittu, Roy Nitulescu, Marjolaine Gauthier-Loiselle, Nicholas Sicignano, Elizabeth Butts, Eric Q. Wu, Brian Barnett

**Affiliations:** Washington Cancer Institute, MedStar Washington Hospital Center, 110 Irving Street, NW, Room C-2149, Washington, DC 20010-2975 USA; Virginia Oncology Associates, Virginia Beach, VA USA; Genentech, Inc., South San Francisco, CA USA; Analysis Group, Inc., Montreal, QC Canada; Health ResearchTx, Trevose, VA USA; Navy and Marine Corps Public Health Center, Portsmouth, VA USA; Analysis Group, Inc., Boston, MA USA

**Keywords:** HER2-positive breast cancer, Trastuzumab, Relapse, Overall survival

## Abstract

**Purpose:**

The NSABP Trial B-31 and NCCTG Trial N9831 (B-31/N9831 trials, Romond et al. in N Engl J Med 353:1673–84, 2005. doi:10.1056/NEJMoa052122; Perez et al. in J Clin Oncol 32:3744–52, 2014. doi:10.1200/JCO.2014.55.5730) established the efficacy of adjuvant trastuzumab for patients with HER2-positive early stage breast cancer. We aimed to estimate the overall survival (OS) and relapse-free survival (RFS) of HER2-positive non-metastatic breast cancer patients treated with adjuvant trastuzumab in a clinical practice setting in the United States.

**Methods:**

Adult women initiating adjuvant trastuzumab within 1 year of breast cancer surgery were identified in the health claims database of the US Department of Defense (01/2003–12/2012). OS and RFS unadjusted rates at 4 and 6 years after the first trastuzumab treatment following the breast cancer diagnosis were estimated from Kaplan–Meier analyses.

**Results:**

The study sample included 3188 women followed for a median of 3.3 years after trastuzumab initiation and treated continuously with trastuzumab for a median of 12 months. The OS rates (95 % confidence intervals) at 4 and 6 years were 90.0 % (88.6–91.2) and 87.1 (85.3–88.6), respectively. The corresponding RFS rates were 75.8 % (74.0–77.5) and 72.7 (70.7–74.7), respectively. The OS and RFS rates at 6 years reported in the B-31/N9831 trials were 89.8 and 81.4 %, respectively.

**Conclusions:**

OS rates estimated in this study were in range with those estimated in the B-31/N9831 trials, while RFS rates were lower. However, patients in the B-31/N9831 trials were younger and possibly had fewer comorbidities than patients in the current study; these differences were not adjusted for in the crude OS and RFS analyses.

**Electronic supplementary material:**

The online version of this article (doi:10.1186/s40064-016-2008-9) contains supplementary material, which is available to authorized users.

## Background

Most patients with breast cancer in developed countries are diagnosed in the early stages of disease (National Cancer Institute 2014; Sant et al. 2003; Allemani et al. 2013; Walters et al. 2013) and undergo tumor-removing surgery, followed by adjuvant chemotherapy and/or targeted therapies against specific overexpressed receptors (van Herk-Sukel et al. 2013; National Comprehensive Cancer Network 2015). Approximately 20 % of the breast cancers are HER2-positive (Parise et al. 2009) and have higher risk of cancer relapse and overall poor prognosis (Esteva and Hortobagyi 2004; Esteva et al. 2002; Parkin et al. 2002). In 2006, the FDA approved trastuzumab, a monoclonal antibody against the HER2 receptor, as adjuvant treatment for HER2-positive breast cancer. The approval was based on the results of the landmark National Surgical Adjuvant Breast and Bowel Project Trial B-31 and the North Central Cancer Treatment Group Trial N9831 (B-31/N9831trials), which have shown that adding trastuzumab to adjuvant chemotherapy reduces the relapse risk by half and improves patient survival (Valabrega et al 2007; Romond et al. 2005; Perez et al. 2014). Subsequent clinical trials reached similar conclusions (Romond et al. 2005; Perez et al. 2014; Gianni et al. 2011; Slamon et al. 2011; Fountzilas et al. 2014; Joensuu et al. 2006; Burstein et al. 2012). However, clinical trials often exclude patients with various comorbidities (Van de Water et al. 2014; Thürmann 2009), leading to estimates that might not always be generalizable to the general patient population (Thürmann 2009; Rothwell 2005). The effectiveness of trastuzumab as adjuvant treatment for HER2-positive breast cancer has also been studied in observational studies (Zurawska et al. 2013; Webster et al. 2012; Peterson et al. 2014; Palmieri et al. 2011; Jensen et al. 2012; Inwald et al. 2014; Hayashi et al. 2013; Bonifazi et al. 2014; Vici et al. 2014). However, these studies faced important challenges: although claims-based studies had large samples, they lacked accurate measures of patient clinical characteristics, including cancer relapse (Zurawska et al. 2013; Webster et al. 2012; Palmieri et al. 2011; Kirby et al. 2010); on the other hand, observational hospital-based studies included detailed clinical information, but were of limited sample size (Jensen et al. 2012; Inwald et al. 2014; Bonifazi et al. 2014) and may have had limited representativeness (Zurawska et al. 2013; Peterson et al. 2014; Palmieri et al. 2011; Hayashi et al. 2013; Vici et al. 2014). Furthermore, to our knowledge, only one of these observational studies was US-based (Hayashi et al. 2013).

The current study aimed to estimate the overall survival (OS) and the relapse-free survival (RFS) of patients with HER2-positive non-metastatic breast cancer treated with adjuvant trastuzumab in a US clinical setting and to compare this information with that observed in the pivotal B-31/N9831trials. To this end, an algorithm to identify breast cancer relapses in administrative claims data was developed and validated against clinical data extracted from a US breast cancer registry.

## Methods

### Data sources

The study used US Department of Defense (DOD) de-identified patient data, covering records from 01/01/2003 to 12/31/2012, obtained from the Military Health System (MHS). MHS contains comprehensive health-related data (inpatient and outpatient healthcare service records, prescription records, and demographic information) on over 9.5 million beneficiaries, including DOD service members, retirees, and their dependents (Guide for DoD Researchers on Using MHS Data 2012). Data were collected from the MHS Data Repository (MDR) claims and clinical database, which includes the Defense Enrollment Eligibility Reporting System (DEERS), and the DOD Automated Central Tumor Registry (ACTUR) (see Additional file 1: Supplemental Methods).

### Selection of study samples

**Fig. 1:**
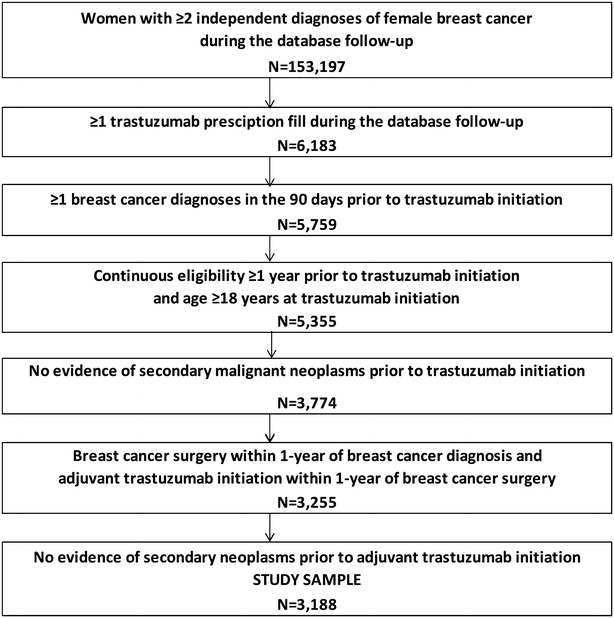
Sample selection flowchart. *Note* The following international classification of diseases, ninth revision, clinical modification (ICD-9-CM) codes were used to identify female breast cancer: 174.x for primary female breast cancer and codes 196.x-199.x for secondary neoplasm, excluding 196.0, 196.1, 196.3 and 198.2, which may be used to indicate locally advanced breast cancer

Adult women receiving trastuzumab alone or in combination with other drugs as adjuvant therapy for non-metastatic breast cancer were selected from the MDR database based on criteria described in Fig. 1 (study sample). Because ACTUR records contain clinical information on breast cancer relapse, the subset of patients from the study sample that had records in the ACTUR (registry sub-sample) was selected to validate the study’s relapse algorithm.

### Outcomes and statistical analyses

#### OS and RFS

The study outcomes were OS and RFS following trastuzumab initiation after breast cancer diagnosis (index trastuzumab, either adjuvant or neoadjuvant). OS and RFS were analyzed in the study sample and the registry sub-sample using time-to-event methods; event time was defined as the time between the index trastuzumab and death from any cause for OS, and relapse or death from any cause for RFS. Patients’ observation periods were censored if they reached the end of the study period (12/31/2012) or lost continuous healthcare coverage without a relapse and/or death event. Date of death was extracted from DEERS via the MDR. An algorithm was developed (described below) to identify relapses in claims data.

OS and RFS rates at 3, 4, 5 and 6 years after index trastuzumab were extracted from the Kaplan–Meier survival curves for the study sample and registry sub-sample. The Kaplan–Meier survival curves from the B-31/N9831trials were extracted for comparison purposes using the GetData Graph Digitizer software (Fedorov 2014) from Perez et al. (2014) and Romond et al. (2012).

#### Relapse algorithm: definition and validation

The algorithm developed to identify relapses in claims data was adapted from previously published algorithms (Chubak et al. 2012; Cheng et al. 2012) to the specifics of adjuvant trastuzumab treatment in non-metastatic breast cancer (see Additional file 1: Supplemental Methods). The patient follow-up was divided in two periods: Period 1, corresponding to the recommended 52-week trastuzumab treatment course (Genentech 2014) (after excluding temporary interruptions in trastuzumab use due to recovery from surgery and/or gaps of <90 days between trastuzumab infusions); and Period 2, corresponding to the remaining follow-up. Four indicators were used to identify relapses in the algorithm: (1) ≥2 independent diagnoses of secondary neoplasm (see Fig. 1 for definition) that were <60 days apart, either in Period 1 or 2; (2) chemotherapy restart in Period 1 after a gap of ≥90 days in treatment; (3) chemotherapy or trastuzumab restart at any time in Period 2; and (4) hormonal therapy initiation in Period 2 for patients who did not use hormonal therapy in Period 1. In some rare cases, these four indicators may signal the presence of a new primary cancer (i.e., new cancer that is not a metastasis of the breast cancer). To avoid counting new primary cancers as breast cancer relapses, the four indicators above were discarded if they were preceded by a new primary cancer diagnosis (i.e., ICD-9-CM 140.x-209.x, excluding codes for breast cancer and/or secondary neoplasm). Additionally, indicators based on one chemotherapy or trastuzumab treatment were discarded if they were not followed by second infusion/injection/prescription fill within 30 days of the chemotherapy or trastuzumab restart. The relapse date corresponded to the date of the earliest observed indicator.

This algorithm was applied to the claims records of the patients in the registry sub-sample and its results were compared with the known relapse status recorded in ACTUR (Additional file 1: Table S1). Three validation statistics were computed: (1) the percentage of patients in the registry sub-sample for whom the claims-based algorithm correctly classified the patients’ relapse status; (2) the sensitivity of the claims-based algorithm, defined as the probability of correctly identifying a true (registry-based) relapse; and (3) the specificity of the claims-based algorithm defined as the probability of correctly identifying a true non-relapse.

#### Other statistical analyses

Patient characteristics were described for the study sample and the registry sub-sample; because the registry sub-sample was a subset of the study sample, no statistical comparisons were conducted between the study sample and the registry sub-sample. All analyses used a two-sided p-value of 0.05 to determine statistical significance. All analyses were performed with SAS v.9.3 (Cary, NC) software.

## Results

### Patient characteristics

The study sample included 3188 patients, observed for a median of 3.3 years after the index trastuzumab. Mean age was 62.1 years (median 63.0) and mean Charlson comorbidity index (CCI) was 4.2 (median 3.0). The most common comorbidities were hypertension, deficiency anemias, valvular heart disease, diabetes, chronic pulmonary disease, hypothyroidism, and depressive disorders (Table 1). Following breast cancer diagnosis, 54.8 % of patients had breast-removing surgery and 45.2 % had breast-conserving surgery. Before initiating adjuvant trastuzumab, 58.0 % of patients received radiation therapy and 8.7 % received neoadjuvant trastuzumab. The median duration of continuous adjuvant trastuzumab treatment was 12 months. For 73.5 % of patients, trastuzumab was initiated ≤6 months post-diagnosis (data not shown). The most common adjuvant regimens in the study sample were TCH-like (28.4 %) and ACTH-like (24.2 %) regimens, trastuzumab monotherapy (19.4 %), and trastuzumab–taxane combinations (14.3 %) (Additional file 1: Table S2).

**Table 1:** Characteristics of the study sample versus registry sub-sample at the initiation of trastuzumab treatment

	Study sample	Registry sub-sample
	N = 3188	N = 624
Demographic characteristics^a^		
Age^b^ [years; mean ± SD (median)]	62.1 ± 12.7 [63.0]	54.3 ± 11.6 [54.0]
Geographic region [N (%)]		
West	771 (24.2)	165 (26.4)
Northeast	607 (19.0)	151 (24.2)
Southeast	791 (24.8)	117 (18.8)
Central	688 (21.6)	103 (16.5)
Other	85 (2.7)	51 (8.2)
Unknown	246 (7.7)	37 (5.9)
Trastuzumab initiation [N (%)]		
Trastuzumab initiated as neoadjuvant therapy^c^	277 (8.7)	50 (8.0)
Trastuzumab initiated as adjuvant therapy^d^	2911 (91.3)	574 (92.0)
Type of surgery prior to adjuvant trastuzumab [N (%)]		
Breast-removing	1746 (54.8)	367 (58.8)
Breast-conserving	1442 (45.2)	257 (41.2)
Radiation therapy prior to the first adjuvant trastuzumab [N (%)]	1849 (58.0)	427 (68.4)
Comorbidities^e^ (>5 % prevalence)		
Charlson comorbidity index (Quan et al. 2005)^f^ (mean ± SD [median])	4.2 ± 2.3 [3.0]	3.7 ± 2.0 [3.0]
Physical comorbidities (Elixhauser et al. 2004)^g^ [N (%)]		
Valvular disease	616 (19.3)	65 (10.4)
Peripheral vascular disorder	203 (6.4)	11 (1.8)
Hypertension, uncomplicated	1681 (52.7)	233 (37.3)
Hypertension, complicated	218 (6.8)	21 (3.4)
Other neurological disorders	184 (5.8)	15 (2.4)
Chronic pulmonary disease	563 (17.7)	65 (10.4)
Diabetes without chronic complications	595 (18.7)	74 (11.9)
Diabetes with chronic complications	176 (5.5)	17 (2.7)
Hypothyroidism	542 (17.0)	75 (12.0)
Rheumatoid arthritis/collagen vascular diseases	166 (5.2)	23 (3.7)
Obesity	371 (11.6)	64 (10.3)
Fluid and electrolyte disorders	400 (12.5)	74 (11.9)
Deficiency anemia	755 (23.7)	147 (23.6)
Psychoses	163 (5.1)	20 (3.2)
Mental comorbidities (American Psychiatric Association 2013)^h^ [N (%)]		
Depressive disorders	502 (15.7)	85 (13.6)
Anxiety disorder	437 (13.7)	85 (13.6)
Trauma- and stressor-related disorders	135 (4.2)	49 (7.9)
Sleep-wake disorders	390 (12.2)	68 (10.9)
Substance-related and addictive disorders	348 (10.9)	60 (9.6)
Cancer characteristics (recorded in cancer registry)		
Breast cancer stage^i^ [N (%)]		
Stage 0	–	1 (0.2)
Stage I	–	260 (41.7)
Stage II	–	268 (42.9)
Stage III	–	85 (13.6)
Stage IV	–	3 (0.5)
Missing	–	7 (1.1)
Tumor size		
Mean size ± SD [median] (mm)	–	28.5 ± 49.6 [20.0]
Missing [N (%)]	–	33 (5.3)
Tumor type [N (%)]	–	
Infiltrating duct carcinoma NOS—invasive	–	454 (72.8)
Infiltrating ductal carcinoma—invasive	–	40 (6.4)
Infiltrating duct carcinoma NOS	–	18 (2.9)
Infiltrating duct and lobular carcinoma—invasive	–	30 (4.8)
Inflammatory carcinoma—invasive	–	11 (1.8)
Lobular carcinoma NOS—invasive	–	12 (1.9)
Infiltrating duct mixed with other types—invasive	–	10 (1.6)
Carcinoma NOS—invasive	–	8 (1.3)
Other (<1 % each)		41 (6.6)
Tumor histologic grade [N (%)]	–	
Grade 1	–	30 (4.8)
Grade 2	–	205 (32.9)
Grade 3	–	330 (52.9)
Grade 4	–	4 (0.6)
Missing	–	55 (8.8)
Lymph node status [N (%)]	–	
No regional lymph node involvement or isolated tumor cells	–	324 (51.9)
Some lymph node involvement	–	272 (43.6)
No regional lymph node involvement, but isolated tumor cells	–	11 (1.8)
Missing	–	17 (2.7)

^a^All demographic characteristics were measured as of the index date, defined as the date of the first trastuzumab

^b^Age groups were based on year of birth

^c^Neoadjuvant therapy was defined as the initiation of trastuzumab alone or in combination with other drugs prior to the breast surgery. By design, patients treated with neo-adjuvant trastuzumab re-initiated trastuzumab following breast surgery

^d^Adjuvant trastuzumab therapy was defined as treatment with trastuzumab alone or in combination with other drugs within 1 year of the breast cancer surgery

^e^Comorbidities and risk factors were measured during the 365 days prior to index trastuzumab

^f^The Charlson comorbidity index (CCI) score includes two points for the cancer diagnosis. Please see corresponding reference

^g^Only comorbidities with >1 % prevalence in both the study sample and registry sub-sample are presented. Please see corresponding reference

^h^Only comorbidities with >1 % prevalence in both the study sample and registry sub-sample are presented. Please see corresponding reference

^i^The charts of 120 patients with missing cancer stage in the Automated Central Tumor Registry (ACTUR) were pulled from the US Department of Defense (DOD) military facilities that followed the patients and were individually reviewed by EB to assign a stage; for 3 patients in this group the pathological report was not available in the patient chart, so the stage was determined clinically; for 7 patients there was not enough information in the patient chart, so the cancer stage remains missing (for patients who receive care in both military and civilian facilities, only the patient charts from the military facilities were available for review)

The registry sub-sample included 624 patients. Mean age was 54.3 years (median 54.0) and mean CCI was 3.7 (median 3.0). Common comorbidities in the registry sub-sample included hypertension and deficiency anemias (Table 1). Most patients in the registry sub-sample initiated trastuzumab as adjuvant therapy (92.0 %), 58.8 % had breast-removing surgery, 41.2 % had breast-conserving surgery, and 68.4 % had radiotherapy prior to the initiation of adjuvant trastuzumab (Table 1). Among patients with available information, 84.8 % had stage 0-II breast cancer and 52.9 % of patients had grade 3 cancer. The mean tumor size was 28.5 mm (median 20.0 mm).

### Relapse algorithm validation

Relapse status was correctly identified in the registry sub-sample in >90 % of cases. The claims-based algorithm had 89.7 % sensitivity and 90.1 % specificity (Table 2).

**Table 2:** Validation of the algorithm used to identify relapses in claims data

	Relapses identified in the ACTUR (cancer registry)			Relapse algorithm validation statistics
	Relapse	No relapse	Total	
Relapses identified in the claims data with the claims based algorithm				
Relapse	True positive N = 26	False positive N = 59	85	90.1 % relapses correctly identified
No relapse	False negative	True negative	539	89.7 % sensitivity
	N = 3	N = 536		90.1 % specificity
Total	29	595	624	

### Description of the relapses identified in the study sample

The algorithm identified 564 (17.7 %) relapses in the study sample. Of these, 216 (38.3 %) occurred within a year of the first adjuvant trastuzumab infusion, 279 (49.5 %) occurred within 1–3 years, and 69 (12.2 %) occurred >3 years later. Among the patients who relapsed within a year of first adjuvant trastuzumab, 30.5 % deaths were observed over a median follow-up of 2.1 years; among those who relapsed within 1–3 years, there were 34.4 % deaths over 1.7 years; finally, among those who relapsed >3 years after the first adjuvant trastuzumab, there were 20.3 % deaths over 1.5 years.

### OS and RFS

**Fig. 2:**
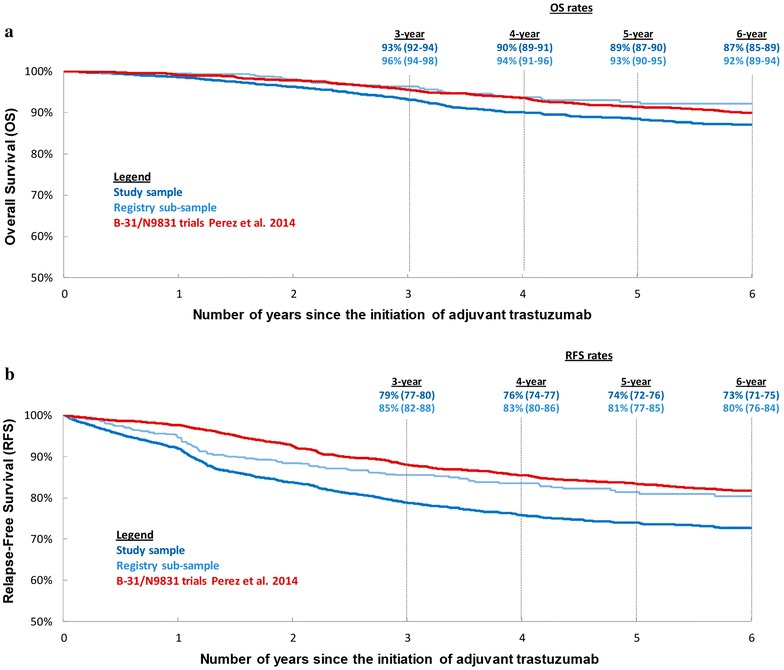
Overall survival and relapse-free survival a in clinical setting versus the B-31/N9831 trials (data from Perez et al. JCO 2014 (Perez et al. 2014) were reproduced with author permission). **a** Overall survival. **b** Relapse-free survival

In the study sample, the unadjusted OS rates were 93.2 % (95 % CI 92.1–94.2 %) at 3 years, 90.0 % (88.6–91.2) at 4 years, 88.5 % (86.9–89.8) at 5 years, and 87.1 % (85.3–88.6) at 6 years; in the registry sub-sample, these rates were 96.3 % (94.3–97.7), 93.7 (91.0–95.6), 92.6 % (89.6–94.8), and 92.2 % (89.1–94.5), respectively (Fig. 2a); finally, the unadjusted OS rate at 6 years in the B-31/N9831trials (Perez et al. 2014) was 89.8 % (Perez et al. 2014; Romond et al. 2012).

In the study sample, the unadjusted RFS rates were 78.8 (77.1–80.3) at 3 years, 75.8 % (74.0–77.5) at 4 years, 73.9 % (72.0–75.7) at 5 years and 72.7 % (70.7–74.7) at 6 years; in the registry sub-sample these rates were 85.5 % (82.2–88.2), 83.5 % (80.0–86.5), 81.4 % (77.5–84.7), and 80.4 % (76.3–83.9), respectively (Fig. 2b); finally, the unadjusted RFS rate at 6 years in the B-31/N9831trials (Perez et al. 2014) was 81.4 % (Perez et al. 2014; Romond et al. 2012).

## Discussion

Because clinical trials recruit patients based on specific selection criteria that may differ from physicians’ treatment decisions in clinical practice, it is important to measure treatment patterns and outcomes in a large clinical setting and see how they compare to those measured in clinical trials. In this retrospective observational study of a large sample of American women with non-metastatic HER2-positive breast cancer receiving adjuvant trastuzumab, a claims-based algorithm was developed to identify breast cancer relapses. The algorithm was validated against known relapse status recorded in ACTUR and found to perform with high specificity (90.1 %) and sensitivity (89.7 %). When applied to the study sample, the algorithm estimated OS rates that were generally similar to those observed in the landmark analysis of the B-31/N9831.trials (Fig. 2a; at 6 years 87.1 % in the current study versus 89.8 % in the B-31/N9831 trials) (Perez et al. 2014), although the RFS rates in the current study were slightly lower than those reported in the B-31/N9831trials (Fig. 2b; at 6 years 72.7 % in the current study versus 81.4 % in the B-31/N9831 trials) (Perez et al. 2014).

Differences in patient characteristics between the current study and the B-31/N9831 trials may explain the observed OS and RFS variations. Patients in the B-31/N9831 trials were generally younger than those in the current study sample and registry sub-sample (Additional file 1: Table S3). However, the age difference is expected to have impacted the observations from the current study more in terms of associated age-related comorbidities and potential tolerability issues (Freedman et al. 2014) than in terms of the intrinsic prognostic value of age on treatment outcomes. While prior studies have shown that younger age is associated with poorer breast cancer prognosis (Freedman et al. 2014), further research revealed that the molecular subtypes of the cancer in question (e.g., HER2-positive) drove this effect of age (Anders et al. 2011), with no age correlation being observed in HER2-positive patients (Partridge et al. 2013). In addition to age differences, patients in the B-31/N9831 trials also tended to have a lower comorbidity profile, as the B-31/N9831 trials excluded patients with significant cardiac diseases, congestive heart failure, or uncontrolled hypertension. Finally, all patients in the trastuzumab arm of the B-31/N9831 trials were treated with ACTH-like treatment regimens, while patients with ACTH-regimens represented only one quarter of the current study’s sample (Romond et al. 2005; Perez et al. 2014).

When analyzing RFS in the registry sub-sample, which was more comparable to the B-31/N9831trials (Perez et al. 2014) sample in terms of age, comorbidity profile, and treatment regimens (35.1 % of the patients in the registry sub-sample were treated with ACTH-like regimens, data not shown), the estimates of RFS were more similar: the RFS at 6 years was 80.4 % for the registry sub-sample versus 81.4 % (Perez et al. 2014) in the B-31/N9831 trials sample (Perez et al. 2014).

The findings of the current study were, however, very similar to those of the HERA trial (Gianni et al. 2011), which reported 4-year OS rate of 89.3 % and RFS rate of 78.6 % among 1703 surgically-treated patients with HER2-positive early stage invasive breast cancer who were assigned to receive 1-year adjuvant trastuzumab after completion of ≥4 cycles of neoadjuvant and/or adjuvant chemotherapy. As in the current study, patients in the trastuzumab arm of the HERA trial were exposed to a variety of neo(adjuvant) therapies prior to the initiation of trastuzumab, with only 26 % receiving neo(adjuvant) chemotherapy with anthracyclines and taxanes (i.e., ACTH-like regimens) (Gianni et al. 2011). Despite the fact that the trastuzumab-treated patients in the HERA trial had lower RFS rates than that of the trastuzumab-treated patients in the B-31/N9831 trials (Perez et al. 2014) and the BCIRG 006 trial (Slamon et al. 2011), their RFS rate at 4 years was significantly higher than that of the control chemotherapy-only arm of the HERA trial (72.2 %, *p* < 0.0001) (Gianni et al. 2011). Based on these findings, it can be hypothesized that some of the patients not treated with standard trastuzumab-based regimens may have poorer outcomes, either because these treatments are less effective than the standard trastuzumab-based treatments or because of underlying conditions that prevent them from receiving the standard treatment.

Observational studies that attempted to estimate OS and RFS in different clinical settings in the US (Hayashi et al. 2013), UK (Webster et al. 2012; Palmieri et al. 2011), Canada (Zurawska et al. 2013; Peterson et al. 2014), Germany (Inwald et al. 2014), Italy (Bonifazi et al. 2014; Vici et al. 2014), and Denmark (Jensen et al. 2012) have shown a large variation in RFS for trastuzumab-treated patients with HER2-positive non-metastatic breast cancer, with 3-year RFS rates ranging from 79.4 % (Bonifazi et al. 2014) to 90.3 (Webster et al. 2012), and 4-year rates ranging from 60 % (Hayashi et al. 2013) to 75.0 % (Bonifazi et al. 2014). Notwithstanding differences that could be driven by variations in methodology, patient characteristics, or care provided by different healthcare systems, the RFS estimates from this study were within the previously reported ranges.

In this study we observed proportions of patients who had mastectomy (55 %; the benchmark proposed by the America College of Surgeons/National Accreditation Program for Breast Centers for the general population of patients with breast cancer is <50 % (American College of Surgeons 2014)) or received adjuvant trastuzumab as monotherapy (20 %) that were slightly higher than expected. These findings may be explained by the overall breast cancer profile of the study sample that may be worse than that of the overall population of patients with non-metastatic breast cancer. Indeed, all patients included in the current study received adjuvant trastuzumab following breast cancer (i.e., were HER2-positive), and a large proportion had large tumors of >2 cm, which make breast conservation surgery more difficult (48 % of registry sub-sample, where tumor size information was available). Also, many of the patients treated with adjuvant trastuzumab monotherapy received neoadjuvant chemotherapy/targeted therapy prior to surgery (49.9 % among those treated with trastuzumab monotherapy versus 13.8 % only in the full study sample) and/or with hormonal therapy prior to the initiation of trastuzumab (18.6 vs. 8.6 %). Interestingly, the patients treated with adjuvant trastuzumab monotherapy also had very high rates of mastectomy, comparable to those observed in the B-31/N9831 trials (63.1 % among those treated with trastuzumab monotherapy vs. 61.4 % in the B-31/N9831 trials (Perez et al. 2014)). These findings suggest that the patients treated with adjuvant trastuzumab monotherapy may have had a different breast cancer profile or different treatment preferences.

The strengths of this study include the large, nationally representative patient sample, the relatively long follow up (median follow-up of 3.3 years), and the use of a validated customized claims-based algorithm to identify relapses among breast cancer patients treated with adjuvant trastuzumab. Of note, the true specificity of our relapse algorithm is likely to be higher than that reported in the current study, because patients that transfer from military to civilian healthcare facilities are lost to registry follow-up, leading to an underestimation of relapses in ACTUR.

This study was also subject to several limitations. First, general limitations of claims data include possible inaccuracies in coding diagnoses, procedures, or pharmacy claims. Second, because patient-level data from the B-31/N9831trials (Perez et al. 2014) is not publicly available, no adjustments were made for patient characteristic differences when comparing the findings from the current study versus those of the B-31/N9831 trials; therefore, RFS variability among studies may be due to this factor. Third, although the relapse algorithm performed well, misclassification of some relapses may have occurred if treatment-based relapse indicators reflected unusual individual treatment patterns rather than the initiation of a treatment post-relapse. For example, or a transient decline in the left ventricle ejection fraction may result in gaps of >90 days in trastuzumab treatment (without relapse). Military families move duty stations every few years and transfer of care from one medical facility to another could cause gaps in trastuzumab treatment (without relapse). Such an occurrence could have resulted in a lower a RFS estimate in the current study. Finally, despite our efforts to exclude all patients with metastatic breast cancer at baseline, some patients with metastatic breast cancer may have been included in the study sample. However, only 0.5 % of the patients in the registry sub-sample had stage IV breast cancer at diagnosis, suggesting such occurrences are rare in the claims data as well (stage IV cases were not excluded from the registry sub-sample to maintain the consistency with the claims-based study sample).

## Conclusion

This descriptive retrospective study of claims and registry data found that the OS and RFS of HER2-positive breast cancer patients treated with trastuzumab in a clinical practice setting were similar to those previously observed in the B-31/N9831 trials and other randomized and observational studies. The findings from this US based study suggest that adjuvant trastuzumab is an effective treatment option across many patients with non-metastatic breast cancer.

## Additional file

10.1186/s40064-016-2008-9 Additional Details on Data Source and Additional Details on the Definition and Validation of Relapse Algorithm. **Figure S1.** ACTUR codes for the type of first relapse. **Figure S2.** Adjuvant-treatment regimens among in the full study sample. **Figure S3.** Patient characteristics in study sample versus registry sub-sample versus B-31/N9831 trials.
