# Supplementary material for: Survival in patients with non-metastatic breast cancer treated with adjuvant trastuzumab in clinical practice
Source: Springerplus. 2016 Mar 31;5:395. doi: 10.1186/s40064-016-2008-9 (PMC4816950; doi:10.1186/s40064-016-2008-9)
Supplement: Supplementary file 1 — 10.1186/s40064-016-2008-9 Additional Details on Data Source and Additional Details on the Definition and Validation of Relapse Algorithm. Figure S1. ACTUR codes for the type of first relapse. Figure S2. Adjuvant-treatment regimens among in the full study sample. Figure S3. Patient characteristics in study sample versus registry sub-sample versus B-31/N9831 trials. [file 40064_2016_2008_MOESM1_ESM.docx]

# ADDITIONAL FILE 1

## Table S1. ACTUR codes for the type of first relapse

| **ACTUR code** | **Description** |
| --- | --- |
| 00 | Patient became disease-free after treatment and has not had a recurrence; leukemia in remission. |
| 04 | *In situ* recurrence of an invasive tumor. |
| 06 | *In situ* recurrence of an *in situ* tumor. |
| 10 | Local recurrence and there is insufficient information available to code to 13-17. Recurrence is confined to the remnant of the organ of origin; to the organ of origin; to the anastomosis; or to scar tissue where the organ previously existed. |
| 13 | Local recurrence of an invasive tumor. |
| 14 | Trocar recurrence of an invasive tumor. Includes recurrence in the trocar path or entrance site following prior surgery. |
| 15 | Both local and trocar recurrence of an invasive tumor (both 13 and 14) |
| 16 | Local recurrence of an *in situ* tumor. |
| 17 | Both local and trocar recurrence of an *in situ* tumor. |
| 20 | Regional recurrence, and there is insufficient information available to code to 21-27. |
| 21 | Recurrence of an invasive tumor in adjacent tissue or organ(s) only. |
| 22 | Recurrence of an invasive tumor in regional lymph nodes only. |
| 25 | Recurrence of an invasive tumor in adjacent tissue or organ(s) and in regional lymph nodes (both 21 and 22) at the same time. |
| 26 | Regional recurrence of an *in situ* tumor, NOS. |
| 27 | Recurrence of an *in situ* tumor in adjacent tissue or organ(s) and in regional lymph nodes at the same time. |
| 30 | Both regional recurrence of an invasive tumor in adjacent tissue or organ(s) and/or regional lymph nodes (20-25) and local and/or trocar recurrence (10, 13, 14, or 15). |
| 36 | Both regional recurrence of an *in situ* tumor in adjacent tissue or organ(s) and/or regional lymph nodes (26 or 27) and local and/or trocar recurrence (16 or 17). |
| 40 | Distant recurrence and there is insufficient information available to code to 46-62. |
| 46 | Distant recurrence of an *in situ* tumor. |
| 51 | Distant recurrence of an invasive tumor in the peritoneum only. Peritoneum includes peritoneal surfaces of all structures within the abdominal cavity and/or positive ascitic fluid. |
| 52 | Distant recurrence of an invasive tumor in the lung only. Lung includes the visceral pleura. |
| 53 | Distant recurrence of an invasive tumor in the pleura only. Pleura includes the pleural surface of all structures within the thoracic cavity and/or positive pleural fluid. |
| 54 | Distant recurrence of an invasive tumor in the liver only. |
| 55 | Distant recurrence of an invasive tumor in bone only. This includes bones other than the primary site. |
| 56 | Distant recurrence of an invasive tumor in the CNS only. This includes the brain and spinal cord, but not the external eye. |
| 57 | Distant recurrence of an invasive tumor in the skin only. This includes skin other than the primary site. |
| 58 | Distant recurrence of an invasive tumor in lymph node only. Refer to the staging scheme for a description of lymph nodes that are distant for a particular site. |
| 59 | Distant systemic recurrence of an invasive tumor only. This includes leukemia, bone marrow metastasis, carcinomatosis, and generalized disease. |
| 60 | Distant recurrence of an invasive tumor in a single distant site (51-58) and local, trocar, and/or regional recurrence (10-15, 20-25, or 30). |
| 62 | Distant recurrence of an invasive tumor in multiple sites (recurrences that can be coded to more than one category 51-59). |
| 70 | Since diagnosis, patient has never been disease-free. This includes cases with distant metastasis at diagnosis, systemic disease, unknown primary, or minimal disease that is not treated. |
| 88 | Disease has recurred, but the type of recurrence is unknown. |
| 99 | It is unknown whether the disease has recurred or if the patient was ever disease-free. |

## Table S2. Adjuvant-treatment regimens in the full study sample

| **Adjuvant regimens^a^** | **Users of the adjuvant regimen**  N (% patients in the study sample) | **Use of other treatments prior to first trastuzumab, by regimen**  n (% patients in the regimen) | | |
| --- | --- | --- | --- | --- |
|  |  | **Neoadjuvant chemotherapy** | **Hormonal therapy from surgery up to first trastuzumab** | **Radiation therapy from diagnosis up to first trastuzumab** |
| TCH-like^b^ | 905 (28.39) | 35 (3.9) | 27 (3.0) | 290 (32.0) |
| ACTH-like^c^ | 772 ( 24.22) | 10 (1.3) | 63 (8.6) | 666 (86.3) |
| Trastuzumab alone | 617 (19.35) | 308 (49.9) | 115 (18.6) | 449 (72.8) |
| Trastuzumab / docetaxel | 267 (8.37) | 28 (10.5) | 10 (3.7) | 119 (44.6) |
| Trastuzumab / paclitaxel | 190 (5.96) | 29 (15.3) | 7 (3.7) | 101 (53.2) |
| Trastuzumab / cyclophosphamide / docetaxel | 152 (4.77) | 3 (2.0) | 11(7.2) | 79 (52.0) |
| Trastuzumab / cyclophosphamide/ doxorubicin | 119 (3.73) | 6 (5.0) | 21 (17.6) | 104 (87.4) |
| All other regimens (frequency < 1%) combined | 166 (5.2) | 20 (12.0) | 20 (12.0) | 126 (75.9) |

**Notes:**

a. Adjuvant regimens were reported based on the drugs used by the patient in the period between the breast cancer surgery and up to 28-days after the first adjuvant trastuzumab (excluding hormonal therapy). By design, all adjuvant regimens include trastuzumab.

b. TCH-like regimens were defined as treatments that included ≥1 of each of the following types of drugs: a taxane, trastuzumab and carboplatin, without any anthracycline ; the three most common TCH-like regimens were the following: trastuzumab / carboplatin / docetaxel (n=822), trastuzumab / carboplatin / paclitaxel (n = 45) and trastuzumab / carboplatin / docetaxel / methotrexate/ paclitaxel (n = 13);

c. ACTH-like regimens were defined as those that included ≥1 of the following types of drugs: a taxane, trastuzumab, an anthracycline and a cyclophosphamide; the three most common ACTH-like regimens were the following: trastuzumab / cyclophosphamide / doxorubicin / paclitaxel (n=535), trastuzumab / cyclophosphamide / docetaxel / doxorubicin (n = 137), and trastuzumab / cyclophosphamide / docetaxel / doxorubicin / paclitaxel (n = 28).

## Table S3. Patient characteristics in study sample versus registry sub-sample versus B-31/N9831 trials (data from Perez et al. JCO 2014^13^ were reproduced with author permission)

| **Patient characteristics** | **Study**  **sample**  **N = 3,188** | **Registry**  **sub-sample^c^**  **N = 624** | **B-31/N9831 trials**^13^  **(trastuzumab arm)**  **N = 2,028** |
| --- | --- | --- | --- |
| **Age ≥ 60 years** ^a,b^, N (%) | 1,917 (60.1) | 210 (33.6) | 357 (17.6) |
| **Charlson comorbidity index**^c^, Mean (SD) | 4.22 (2.3) | 3.72 (2.0) | Not available |
| **Selected comorbidities**^c^, N (%) |  |  |  |
| Valvular disease | 616 (19.3) | 65 (10.4) | Excluded if clinically significant |
| Chronic pulmonary disease | 563 (17.7) | 65 (10.4) | Not available |
| Diabetes | 771 (24.2) | 91 (14.6) | Not available |
| Hypothyroidism | 542 (17.0) | 75 (12.0) | Not available |
| Congestive heart failure | 136 (4.3) | 11 (1.8) | Excluded |
| Hypertension | 1,839 (57.7) | 254 (40.7) | Excluded if poorly controlled |
| **Cancer characteristics**^b^, N (%) |  |  |  |
| Tumor size ≥ 5.1 cm | Not available | 46 (7.4) | 195 (9.6) |
| Tumor grade ≥ 3 (poor) | Not available | 334 (53.5) | 1,414 (69.7) |
| Lymph node involvement | Not available | 272 (43.6) | 1,895 (93.4) |
| **Breast-removal surgery** ^a,b^, N(%) | 1,746 (54.8) | 367 (58.8) | 1,246 (61.4) |

**Notes**

a. Differences in patient characteristics between the study sample and the trastuzumab arm in the B-31/N9831 trial were statistically significant at p ≤ 0.05 when tested with Chi-Square tests.

b. Differences in patient characteristics between the registry sub-sample and the trastuzumab arm in the B-31/N9831 trial were statistically significant at p ≤ 0.05 when tested with Chi-Square tests.

c. When the 624 patients from the registry sub-sample (i.e., patients with ACTUR records) were compared with the sub-sample of patients who did not have ACTUR records (2,564 / 3,188), the patients in the registry sub-sample were significantly older (mean age 64.0 vs. 54.3, p<0.001 using Wilcoxon test), had a higher overall comorbidity burden (CCI 4.3 vs. 3.7, p<0.001 using Chi-Square test), and lower prevalence of valvular disease, chronic pulmonary disease, diabetes, hypothyroidism, congestive heart failure, and hypertension (data not shown, all p<0.05, using Chi-square tests)**.**

# Supplemental Methods – Additional Details on Data Source

The MDR includes claims per service data for inpatient and outpatient healthcare services delivered in military and civilian facilities paid for through the TRICARE healthcare plan, as well as demographic information and medication prescriptions for all MHS beneficiaries. In addition, clinical information on tobacco use and alcohol use is available beginning in October 2008 from the electronic medical records (EMRs) of military facilities.

The ACTUR records all cancer cases followed in DOD military facilities. The registry includes information on patient demographics; cancer characteristics at the time of the diagnosis, including stage, grade, tumor size, location and histology, and lymph node status; first-line therapy received, including surgery and chemotherapy; and some information on outcomes that occurred during the treatment, including the type and timing of relapses.

The research data were derived from an approved Naval Medical Center, Portsmouth, VA Institutional Review Board (IRB) protocol and all research was conducted in compliance with federal and state laws, including the Health Insurance Portability and Accountability Act of 1996 (HIPAA).

# Supplemental Methods – Additional Details on the Definition and Validation of Relapse Algorithm

The algorithm to identify relapse in claims data was developed by adapting previously published algorithms to the specifics of adjuvant trastuzumab treatment in non-metastatic breast cancer based on the existing literature and included: an algorithm developed by Chubak et al. [35] that identifies relapses in claims databases through the use of ICD-9-CM diagnostic codes for secondary neoplasms, and another algorithm in which a relapse is assumed to occur for patients reinitiating a cancer treatment within a given period (>3 months) after completion of their index therapy for breast cancer (Dignam et al [36] and Cheng et al [37]).
